# Inhibition of miR‐499a‐5p Ameliorates Apoptotic and Autophagic Damage in Hypoxic Cardiomyocytes H9c2 Through Upregulation of ARGLU1

**DOI:** 10.1002/kjm2.70059

**Published:** 2025-07-22

**Authors:** Sha Wang, Hui‐Jun Wang, Shuo Pan

**Affiliations:** ^1^ Department of Cardiovascular Surgery Shaanxi Provincial People's Hospital Xi'an Shaanxi China; ^2^ Department of General Surgery The First Affiliated Hospital of Xi'an Jiaotong University Xi'an City Shaanxi China

**Keywords:** apoptosis, arginine and glutamate rich 1, autophagy, MiR‐499a‐5p, myocardial infarction

## Abstract

Myocardial infarction (MI), the most prevalent form of acute coronary syndrome, is often accompanied by cardiomyocyte apoptosis. In addition to apoptosis, autophagy plays a critical role in determining cardiomyocyte survival during MI. This study aimed to elucidate the regulatory role of miR‐499a‐5p in cardiomyocyte apoptosis and autophagy under hypoxic conditions. An MI mouse model was established via ligation of the left anterior descending coronary artery, and RT‐qPCR was used to assess miR‐499a‐5p expression levels in cardiac tissues from MI and sham‐operated mice. Masson's trichrome staining was employed to evaluate cardiac fibrosis, and echocardiography was conducted to assess cardiac functional parameters. For in vitro experiments, TUNEL assays and flow cytometry analyses were used to measure apoptosis and autophagy. A luciferase reporter assay confirmed the direct binding between miR‐499a‐5p and arginine and glutamate rich 1 (ARGLU1). Western blot analysis was used to quantify protein levels of apoptotic markers, autophagy‐related proteins, and ARGLU1. The results demonstrated that MI mice developed significant cardiac fibrosis and functional impairment, along with increased miR‐499a‐5p expression. In H9c2 cells, knockdown of miR‐499a‐5p significantly reduced hypoxia‐induced apoptosis and autophagy, whereas miR‐499a‐5p overexpression exacerbated these processes. Moreover, ARGLU1 was identified as a direct target of miR‐499a‐5p and was negatively regulated by it. Silencing ARGLU1 enhanced hypoxia‐induced apoptosis and autophagy and reversed the protective effects observed with miR‐499a‐5p knockdown. In summary, miR‐499a‐5p inhibition mitigates hypoxia‐induced injury in H9c2 cells by reducing apoptosis and autophagy through the upregulation of ARGLU1, suggesting a potential therapeutic target for MI.

## 1 Introduction

Myocardial infarction (MI) is responsible for approximately half of all cardiovascular‐related deaths globally and remains a significant public health concern due to its limited effectiveness in reperfusion therapy [1, 2]. A variety of risk factors, including excessive alcohol consumption, physical inactivity, hypertension, and unhealthy dietary habits, have been implicated in the pathogenesis of MI [3]. The onset of MI triggers a cascade of pathological events, including extensive myocardial apoptosis and fibrosis, heightened inflammatory responses, enlargement of the infarct area, and excessive production of reactive oxygen species (ROS) [4, 5]. Autophagy, a cellular self‐degradation process, has been closely associated with the pathophysiology of several cardiovascular disorders, including MI [6]. While autophagy has been shown to play a cardioprotective role under certain circumstances, other studies have reported its detrimental effects in the context of MI [7, 8]. However, the precise role of autophagy in MI development needs to be further elucidated.

MicroRNAs (miRNAs) are endogenous, short noncoding RNA molecules that regulate gene expression at the post‐transcriptional level by targeting specific messenger RNAs [9]. A growing body of evidence indicates that miRNAs play crucial roles in modulating the pathological mechanisms underlying various cardiac conditions. For instance, miR‐30d is significantly downregulated in myocardial tissues after MI, and its overexpression has been shown to inhibit autophagy in H9c2 cardiomyocytes [10]. Similarly, miR‐106a impairs autophagy and angiogenesis in venous endothelial cells post‐MI by targeting ATG7 [11]. Furthermore, miR‐17‐5p is markedly upregulated in myocardial tissue after MI, and its inhibition has been found to reduce myocardial autophagy as evidenced by decreased Beclin‐1 expression, a lower LC3II/LC3I ratio, and increased p62 levels—thereby alleviating myocardial remodeling [12]. Notably, miR‐499a‐5p was reported as a circulating biomarker of hypertrophic cardiomyopathy [13], and its expression was revealed to be upregulated in patients with hypertrophic cardiomyopathy than healthy controls [14]. In contrast, its expression is markedly downregulated in cases of sepsis‐induced myocardial dysfunction [15]. Zhao et al. further demonstrated a reduction in serum miR‐499a‐5p levels in hypoxia/reoxygenation cell models, and revealed that miR‐499a‐5p exerts a protective effect by alleviating cardiomyocyte injury under such conditions [16]. Additionally, the study by Wang et al. underscored the diagnostic and prognostic potential of miR‐499a‐5p in acute MI [17]. Based on these findings, we hypothesized that miR‐499a‐5p may play a regulatory role in the progression of MI through specific molecular mechanisms.

In this study, a mouse model of MI was established through ligation of the left anterior descending (LAD) coronary artery, a widely accepted method for mimicking the pathophysiological features of human MI. The primary objective was to investigate the functional role and underlying molecular mechanisms of miR‐499a‐5p in hypoxia‐induced injury in H9c2 rat cardiomyocytes, an in vitro model representing myocardial stress conditions. The study provides compelling evidence that miR‐499a‐5p promotes hypoxia‐induced cell damage, while its inhibition exerts protective effects by attenuating both apoptosis and autophagy through the upregulation of arginine‐ and glutamate‐rich 1 (ARGLU1). These findings contribute to a deeper understanding of the molecular events driving cardiomyocyte injury in MI and suggest that targeting miR‐499a‐5p may offer a novel therapeutic strategy for preserving myocardial function and mitigating adverse cardiac remodeling in MI patients.

## 2 Materials and Methods

### 2.1 Animal Model

Male C57BL/6 mice (*n* = 12; 10 weeks old; 20–25 g) were obtained from Cavens Lab Animal Co. Ltd. (Changzhou, China). Animals were housed in a temperature‐controlled environment (23°C ± 2°C) with a 12‐h light/dark cycle and 60% relative humidity. Mice were divided into two groups (*n* = 6 in each group): Sham and MI groups. The MI mouse model was established by performing the ligation of the LAD. Briefly, mice were anesthetized with 3% isoflurane and placed in a supine position for thoracotomy. After the heart was exposed, the LAD was ligated using a 6–0 silk suture. In the sham group, the same surgical procedure was performed except for the LAD ligation. At 7 or 14 days post‐surgery, echocardiography was conducted under anesthesia to evaluate cardiac function, including left ventricular end‐systolic diameter (LVESD), left ventricular end‐diastolic dimension (LVEDD), lung weight to tibia length ratio (LW/TL), and heart weight to tibia length ratio (HW/TL). After these assessments, mice were euthanized, and cardiac tissues were harvested for further analysis. All experimental procedures involving animals were approved by the Institutional Animal Care and Use Committee of Hubei Provincial Center for Disease Control and Prevention (Safety Evaluation Research Center Animal Welfare No. 202320113; July 12th, 2023), in accordance with ethical guidelines.

### 2.2 Masson's Trichrome Staining

To evaluate the extent of cardiac fibrosis, Masson's trichrome staining was performed on cardiac tissue sections. After fixation in 4% paraformaldehyde, the tissue samples were decalcified, dehydrated, cleared with xylene, and subsequently embedded in paraffin. The sections of 5 μm thickness were prepared and stained using Masson's trichrome reagent. The degree of fibrosis was quantified by analyzing the stained sections with ImageJ software.

### 2.3 Cell Culture and Transfection

H9c2 rat cardiomyocytes were obtained from the American Type Culture Collection (ATCC, USA) and maintained in Dulbecco's Modified Eagle Medium (DMEM) supplemented with 10% fetal bovine serum (FBS) and 1% penicillin–streptomycin in a humidified incubator. To establish an in vitro hypoxia model, cells were placed in a hypoxic chamber (containing about 99.9% of N_2_ and less than 1% of O_2_) at 37°C for 72 h. Then, Lipofectamine 2000 (Invitrogen, USA) was used to perform cell transfection. H9c2 cells were transfected with miR‐499a‐5p mimics, miR‐499a‐5p inhibitor, shRNA targeting ARGLU1 (sh‐ARGLU1), or their respective negative controls (NC mimics, NC inhibitor, and sh‐NC) (all from RiboBio, China). Transfection was carried out for 48 h before further experimental analysis.

### 2.4 Terminal Deoxynucleotidyl Transferase dUTP Nick‐End Labeling (TUNEL) Staining

To evaluate apoptosis in H9c2 cardiomyocytes, a TUNEL apoptosis detection kit (Roche, Switzerland) was utilized. Briefly, cells were first fixed with 4% paraformaldehyde (PFA) and then permeabilized using 0.1% Triton X‐100. After rinsing with phosphate‐buffered saline (PBS), the TUNEL reaction mixture (R&D Systems, USA) was added and incubated with the cells for 1 h at room temperature (RT). Subsequently, nuclei were counterstained with 4’,6‐diamidino‐2‐phenylindole (DAPI) obtained from Elabscience, China, for 10 min in the dark. The apoptotic cells were then analyzed using a flow cytometer (Beckman Coulter).

### 2.5 Flow Cytometry

Autophagy levels in H9c2 cardiomyocytes were assessed by flow cytometry using monodansylcadaverine (MDC) staining, as previously described [18]. Briefly, cells were incubated with MDC solution (50 μM; Sigma, USA) for 10 min at room temperature in the dark. After incubation, cells were washed three times with PBS to remove excess dye. The fluorescence intensity was then measured using a flow cytometer (Becton‐Dickinson, USA) to quantify autophagic activity.

### 2.6 Luciferase Reporter Assay

Targetscan system (http://www.targetscan.org/vert_72/) was used to predict the potential targets of miR‐499a‐5p, and ARGLU1 was confirmed as a target downstream of miR‐499a‐5p. To validate the interaction between miR‐499a‐5p and ARGLU1, H9c2 cells (KL237H; Kanglang, Shanghai, China) were seeded into 24‐well plates and cultured for 24 h. The wild‐type (ARGLU1‐WT) and mutant (ARGLU1‐Mut) 3′‐UTR sequences of ARGLU1 were cloned into the pmirGLO luciferase reporter vector (Genscript, China). H9c2 cells were then co‐transfected with either ARGLU1‐WT or ARGLU1‐Mut reporter constructs along with miR‐499a‐5p mimics or negative control (NC) mimics using Lipofectamine 2000 (Invitrogen). After 48 h of transfection, luciferase activity was measured using the Dual‐Luciferase Reporter Assay System (Promega, USA) to assess the direct binding between miR‐499a‐5p and the 3’‐UTR of ARGLU1.

### 2.7 Western Blotting

H9c2 cardiomyocytes were lysed using radioimmunoprecipitation assay (RIPA) buffer (Beyotime, China) supplemented with phenylmethylsulfonyl fluoride (PMSF). Total protein concentrations were determined using a bicinchoninic acid (BCA) assay kit (Beyotime). Equal amounts of protein (30 μg per sample) were separated by 10% sodium dodecyl sulfate‐polyacrylamide gel electrophoresis (SDS‐PAGE) and subsequently transferred onto polyvinylidene fluoride (PVDF) membranes (Solarbio Science & Technology, China). The membranes were blocked with 5% non‐fat milk and then incubated overnight at 4°C with the following primary antibodies: anti‐Cleaved caspase‐3 (DRF10148; 1:1000; Wanwu, China), anti‐p53 (ab131442; 1:500; Abcam, UK), anti‐LC3 II (#2775, 1:1000, Cell Signaling Technology), anti‐LC3I (#4599; 1:1000, Cell Signaling Technology), anti‐p62 (ab109012; 1:10000, Abcam), anti‐ARGLU1 (ARG40881; 1:1000; Arigo, China), and anti‐β‐actin (ab8224; 1:1000, Abcam). After washing with 0.1% Tris‐buffered saline containing Tween‐20 (TBST), the membranes were incubated with appropriate secondary antibodies at room temperature for 1 h. Protein bands were visualized using an enhanced chemiluminescence (ECL) detection system (Thermo Fisher Scientific, USA), and β‐actin was used as a reference for normalization.

### 2.8 Reverse Transcription Quantitative PCR (RT‐qPCR)

Total RNA was extracted from H9c2 rat cardiomyocytes and mouse cardiac tissues using 500 μL of TRIzol reagent (Invitrogen, USA), and RNA was eluted in RNase‐free water. The isolated RNA was then reverse‐transcribed into complementary DNA (cDNA) using the RevertAid First Strand cDNA Synthesis Kit (Thermo Scientific, Waltham, USA). Quantitative PCR was subsequently performed with the QuantiNova SYBR Green PCR Kit (Qiagen, Germany) on a QuantStudio 3 Real‐Time PCR System (Thermo Scientific). GAPDH was used as the internal reference gene for mRNA expression, while U6 served as the endogenous control for miR‐499a‐5p. Relative gene expression levels were calculated using the 2^−ΔΔCt^ method, and the primer sequences used are shown in Table 1.

**TABLE 1:** The primers used in this manuscript.

GENE	Forward primer	Reverse primer
Rat		
MiR‐499a‐5p	GAGTGTGGCGGGGAATCT	GCGTGGTGCAGTCGTGTG
UBE2V2	CCGCTCGAGATGGCGGTCTCCACAG	CGGGATCCTTACAGATCCTCTTCTGAGATG
ARGLU1	GAGCAGCAGAGAAAGGGAGA	CAGCCGAGCACTCTAGCTCT
RAB5C	CCAACACAGATACATTTGCAC	CCAACACAGATACATTTGCAC
OSBPL1A	TCTCTGGTGAAAGGCTCTC	TTCGGACTCGGAATCTGAC
U6	GCTTCGGCAGCACATATACTAAAAT	CGCTTCACGAATTTGCGTGTCAT
GAPDH	GTTGTGGCTCTGACATGCT	CCCAGGATGCCCTTTAGT
Mouse		
α‐SMA	AAACAGGAATACGACGAAG	CAGGAATGATTTGGAAAGGA
MiR‐499a‐5p	GCCCTGTCCCCTGTGCCTT	AAACATCACTGCAAGTCTT
collagen I	AAGAAGACATCCCTGAAGTCA	TTGTGGCAGATACAGATCAAG
U6	CTCGCTTCGGCAGCACA	AACGCTTCACGAATTTGC
GAPDH	AACGCTTCACGAATTTGC	ACTCCATCACCATCTTCCAG

### 2.9 Statistical Analysis

All quantitative data are expressed as mean ± standard deviation (SD). Statistical analyses were performed using SPSS version 22.0 (IBM Corp., USA). One‐way analysis of variance (ANOVA) was applied for comparisons among multiple groups, while Student's t‐test was used for comparisons between two groups. A *p*‐value < 0.05 was considered to indicate statistical significance.

## 3 Results

### 3.1 MiR‐499a‐5p Was Upregulated in Cardiac Tissues of MI Mice

**FIGURE 1:**
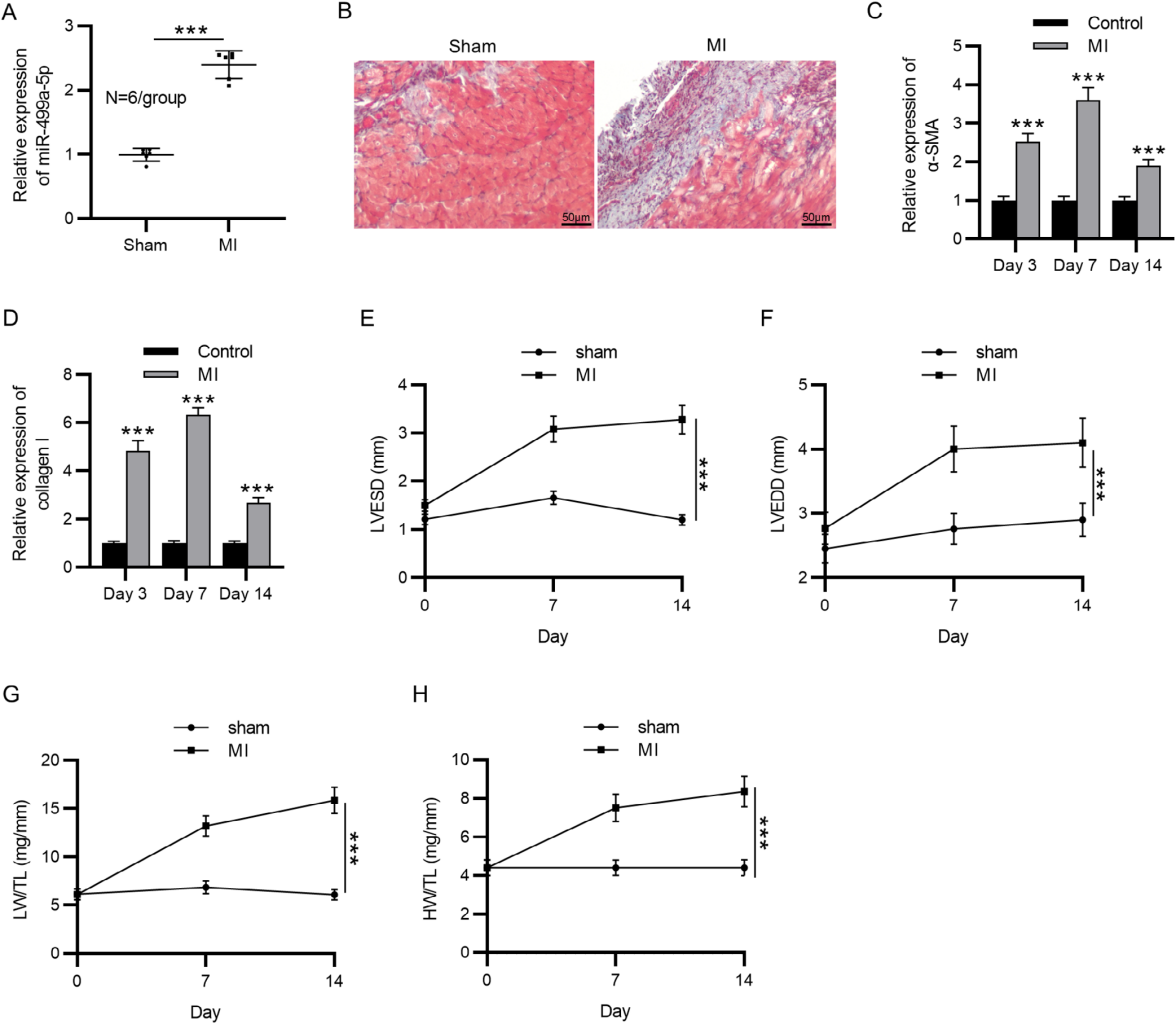
MiR‐499a‐5p was upregulated in cardiac tissues of MI mice. (A) The expression level of miR‐499a‐5p in cardiac tissues from MI and sham‐operated mice was determined using RT‐qPCR. (B) Masson's trichrome staining was performed to evaluate collagen deposition in the cardiac tissue of MI and sham groups. (C–D) RT‐qPCR analysis of fibrotic markers α‐SMA and collagen I was conducted on days 3, 7, and 14 after establishment of MI mouse model to assess temporal changes in fibrosis‐related gene expression. (E–H) Echocardiography analysis was used to measure cardiac function parameters, including left ventricular end‐systolic diameter (LVESD), left ventricular end‐diastolic diameter (LVEDD), lung weight to tibia length ratio (LW/TL), and heart weight to tibia length ratio (HW/TL) on days 3, 7, and 14 post‐MI. Data are presented as mean ± SEM. ***p < 0.001. ***p < 0.001.

After the establishment of the MI mouse model, RT‐qPCR was performed to measure miR‐499a‐5p expression in cardiac tissues of MI mice and sham‐operated mice. As shown in Figure 1A, miR‐499a‐5p levels were significantly elevated in the MI group compared to the sham group. In addition, Masson's trichrome staining revealed marked collagen deposition in the MI group, indicative of increased fibrosis (Figure 1B). The expression of fibrosis‐related markers, including α‐smooth muscle actin (α‐SMA) and collagen I, was significantly upregulated at 3, 7, and 14 days post‐MI, as demonstrated by RT‐qPCR (Figure 1C,D). Furthermore, echocardiographic assessment showed that key indicators of cardiac dysfunction, including LVESD, LVEDD, LW/TL, and HW/TL, in the MI group were significantly higher than those in the control group (Figure 1E–H). Collectively, these results confirm the successful establishment of the MI mouse model and confirm the upregulation of miR‐499a‐5p in cardiac tissues following MI.

### 3.2 Inhibition of miR‐499a‐5p Inhibits Hypoxia‐Induced H9c2 Cell Apoptosis and Autophagy

**FIGURE 2:**
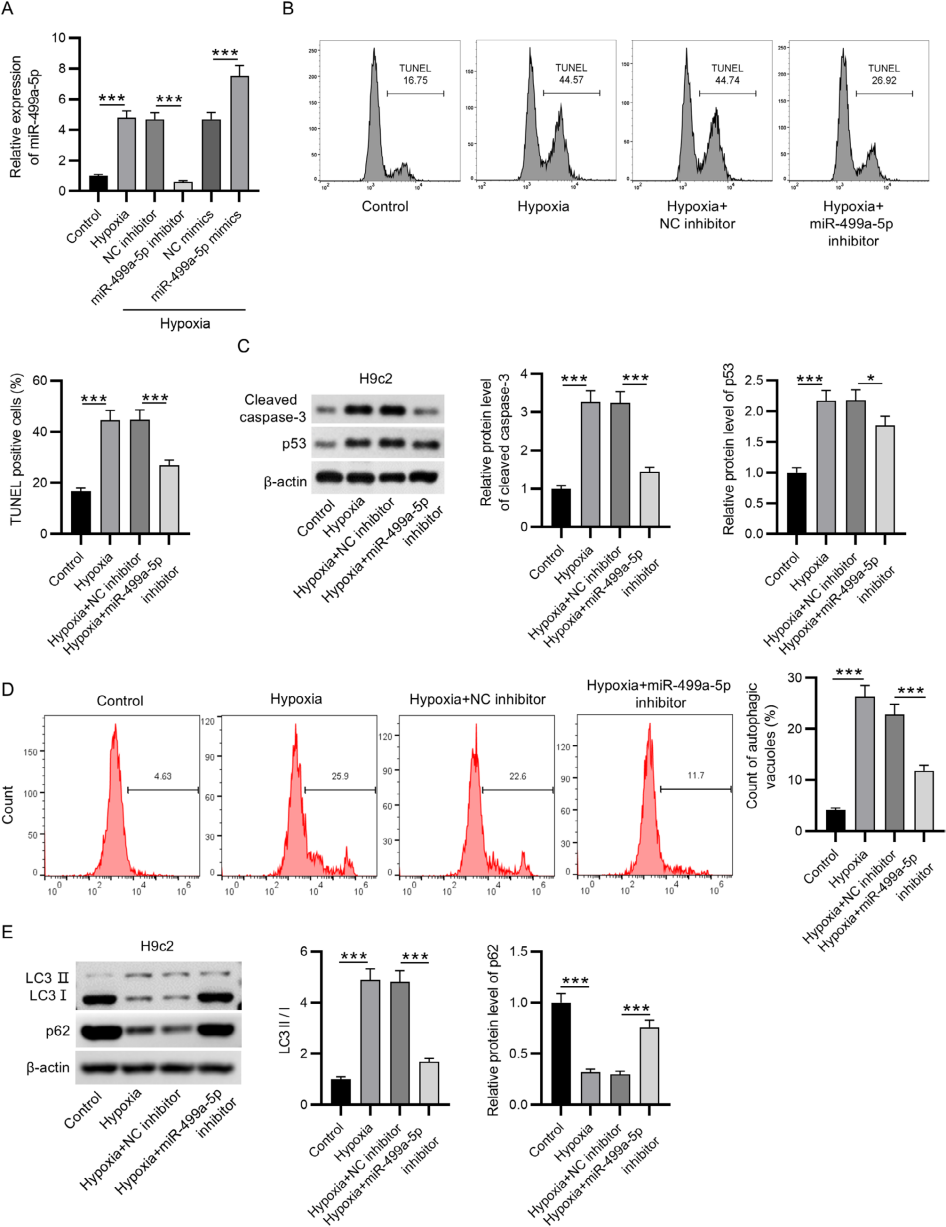
Inhibition of miR‐499a‐5p attenuated hypoxia‐induced H9c2 cell apoptosis and autophagy. (A) RT‐qPCR analysis of miR‐499a‐5p expression was conducted in H9c2 cells under different treatment conditions: Control, hypoxia, hypoxia + negative control (NC) inhibitor, hypoxia + miR‐499a‐5p inhibitor, hypoxia + NC mimics, and hypoxia + miR‐499a‐5p mimics. (B) TUNEL assays were conducted to measure apoptosis in H9c2 cells following the indicated treatments. (C) Western blotting was conducted to evaluate protein levels of apoptotic markers (cleaved caspase‐3 and p53) in H9c2 cells. (D) Flow cytometry was employed to evaluate autophagic vacuole accumulation in H9c2 cells. (E) Western blotting was performed to measure protein levels of LC3I, LC3II, and p62 in H9c2 cells. Data are expressed as mean ± SEM. *p < 0.05, ***p < 0.001.

To establish an in vitro model of MI, H9c2 rat cardiomyocytes were subjected to hypoxic conditions. Consistent with the in vivo findings, miR‐499a‐5p expression was significantly upregulated in hypoxia‐exposed H9c2 cells compared to untreated controls (Figure 2A). To modulate miR‐499a‐5p levels, miR‐499a‐5p inhibitors or mimics were transfected into hypoxia‐stimulated H9c2 cells. As expected, miR‐499a‐5p expression was significantly reduced in the miR‐499a‐5p inhibitor group relative to the NC inhibitor group, whereas cells transfected with miR‐499a‐5p mimics showed a marked increase in expression compared to NC mimics (Figure 2A). TUNEL staining revealed that hypoxia induced apoptosis in H9c2 cells. Notably, knockdown of miR‐499a‐5p significantly reduced the percentage of TUNEL‐positive cells, indicating a protective effect against hypoxia‐induced apoptosis (Figure 2B). Protein expression analysis revealed that the levels of pro‐apoptotic markers, including cleaved caspase‐3 and p53, were significantly elevated in H9c2 cells following hypoxic stimulation, compared to the control group. This apoptotic dysregulation was notably ameliorated by miR‐499a‐5p inhibition (Figure 2C). Additionally, flow cytometry demonstrated a marked accumulation of autophagic vacuoles in response to hypoxia, an effect that was reversed upon miR‐499a‐5p knockdown (Figure 2D). In line with these findings, hypoxia‐induced upregulation of the LC3‐II/LC3‐I ratio and downregulation of p62 protein levels were also significantly restored by miR‐499a‐5p inhibition, indicating suppression of autophagic activity (Figure 2E). Taken together, these results suggest that inhibition of miR‐499a‐5p can effectively attenuate hypoxia‐induced apoptosis and autophagy in H9c2 cardiomyocytes, highlighting its potential as a therapeutic target for MI.

### 3.3 Overexpression of miR‐499a‐5p enhanced hypoxia‐induced H9c2 cell apoptosis and autophagy

**FIGURE 3:**
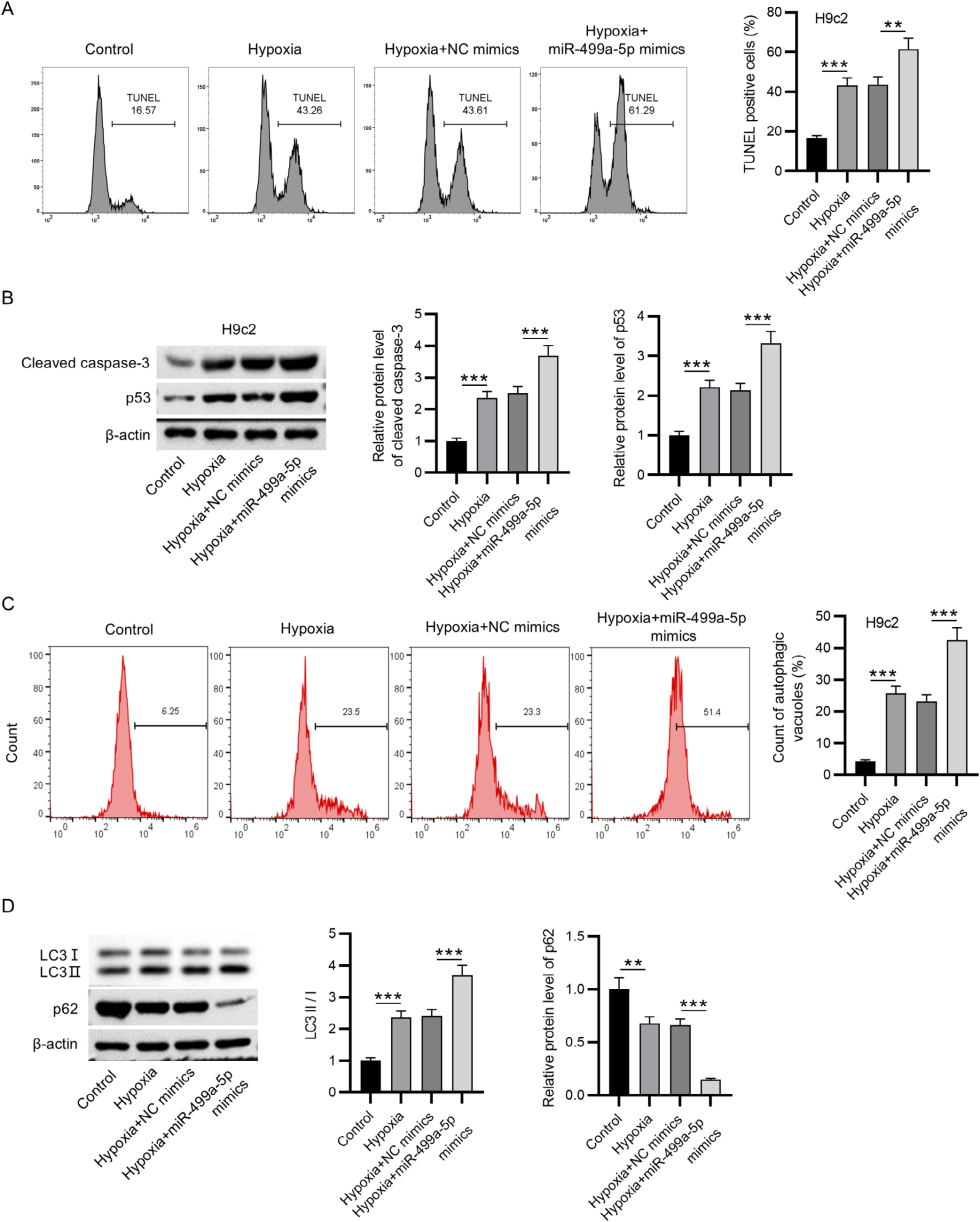
Overexpression of miR‐499a‐5p enhanced hypoxia‐induced H9c2 cell apoptosis and autophagy. (A) TUNEL assays were performed to assess apoptosis in H9c2 cells under normoxic (control), hypoxic, hypoxia + negative control (NC) mimics, and hypoxia + miR‐499a‐5p mimics conditions. (B) Western blot analysis was used to quantify the expression of apoptotic markers in H9c2 cells, with β‐actin serving as the internal loading control. (C) Flow cytometry analysis was conducted to determine the accumulation of autophagic vacuoles in H9c2 cells. (D) Protein levels of autophagic factors (LC3I, LC3II, and p62) were measured by western blotting. Data are presented as mean ± SEM. **p < 0.01, ***p < 0.001.

To elucidate the functional impact of miR‐499a‐5p overexpression on hypoxia‐induced injury in cardiomyocytes, gain‐of‐function experiments were conducted in H9c2 cells. The results, illustrated in Figure 3A, demonstrated a significant increase in TUNEL‐positive cells following hypoxic exposure, an effect that was further amplified by miR‐499a‐5p overexpression, indicating enhanced apoptotic activity. Consistent with this observation, western blot analysis (Figure 3B) revealed that the hypoxia‐induced elevation of cleaved caspase‐3 and p53 protein levels was further upregulated upon overexpression of miR‐499a‐5p. This suggests that miR‐499a‐5p actively contributes to the apoptotic cascade under stress conditions. Moreover, the increased percentage of autophagic vacuoles in the hypoxia group compared to the control group was further promoted by overexpressing miR‐499a‐5p (Figure 3C). This indicates that miR‐499a‐5p not only influences apoptosis but also modulates autophagic processes during hypoxic stress. Further confirmation was obtained from the analysis of key autophagy‐related proteins. As shown in Figure 3D, hypoxia led to an increase in the LC3‐II/LC3‐I ratio, indicative of enhanced autophagosome formation, and a decrease in p62 levels, reflecting autophagic flux. Both these molecular signatures were further intensified by miR‐499a‐5p overexpression, reinforcing the conclusion that miR‐499a‐5p plays a pro‐autophagic role under hypoxic conditions. In summary, hypoxia‐induced H9c2 cell apoptosis and autophagy were enhanced by miR‐499a‐5p overexpression.

### 3.4 ARGLU1 Was Identified as a Potential Target of miR‐499a‐5p

**FIGURE 4:**
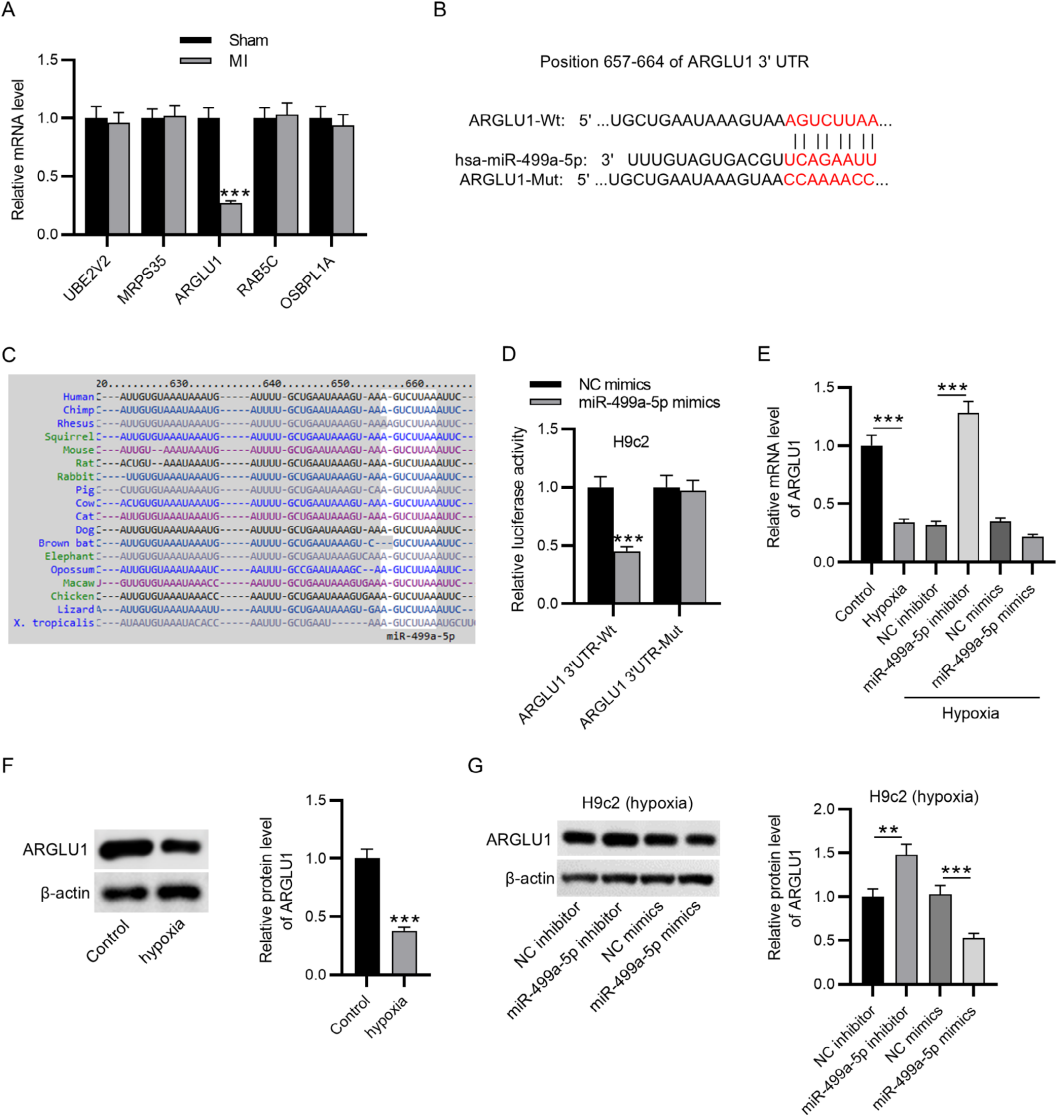
ARGLU1 was identified as a potential target of miR‐499a‐5p. (A) RT‐qPCR was performed to assess the expression levels of the top five predicted miR‐499a‐5p target genes in cardiac tissues from MI and sham‐operated mice. (B) TargetScan was used to predict a conserved binding site between miR‐499a‐5p and ARGLU1. (C) Sequence alignment demonstrated conservation of the miR‐499a‐5p binding site in ARGLU1 across multiple species. (D) Dual‐luciferase reporter assay was conducted to verify the direct interaction between miR‐499a‐5p and ARGLU1. (E) RT‐qPCR was utilized to measure the mRNA expression of ARGLU1 in H9c2 cells under control, hypoxia, NC inhibitor, miR‐499a‐5p inhibitor, NC mimics, and miR‐499a‐5p mimics groups. (F) Western blotting was performed to measure the protein level of ARGLU1 in H9c2 cells with or without hypoxia exposure. (G) Quantification of ARGLU1 protein levels by western blotting in hypoxia‐stimulated H9c2 cells treated with NC inhibitor, miR‐499a‐5p inhibitor, NC mimics, or miR‐499a‐5p mimics. Data are shown as mean ± SEM.**p < 0.01, ***p < 0.001.

TargetScan was used to predict the downstream mRNAs of miR‐499a‐5p, and the top five genes were identified for further analysis. Among these, only ARGLU1 exhibited a significant reduction in expression in cardiac tissues from the MI group compared to the control group (Figure 4A). The binding site of miR‐499a‐5p and ARGLU1 was predicted from TargetScan (Figure 4B), and this binding site was found to be highly conserved across multiple species, including rat, mouse, human, and rabbit (Figure 4C). To validate this interaction, a dual‐luciferase reporter assay was performed. Overexpression of miR‐499a‐5p significantly suppressed luciferase activity in H9c2 cells transfected with the wild‐type construct ARGLU1‐WT, whereas no significant change was observed in cells transfected with the mutant construct ARGLU1‐Mut (Figure 4D), confirming a direct binding interaction between miR‐499a‐5p and ARGLU1. Furthermore, both mRNA and protein levels of ARGLU1 were significantly downregulated in H9c2 cells exposed to hypoxia (Figure 4E,F). Notably, knockdown of miR‐499a‐5p restored ARGLU1 expression in hypoxia‐treated cells, as compared to the hypoxia + NC inhibitor group (Figure 4E,G). Conversely, miR‐499a‐5p overexpression led to a marked reduction in ARGLU1 expression under hypoxic conditions when compared to NC mimics (Figure 4E,G). The findings demonstrate that ARGLU1 levels were negatively regulated by miR‐499a‐5p in H9c2 cells under hypoxia, suggesting a direct post‐transcriptional regulatory mechanism by which miR‐499a‐5p may exacerbate myocardial injury.

### 3.5 Knockdown of ARGLU1 Promoted Hypoxia‐Induced Cell Apoptosis and Autophagy

**FIGURE 5:**
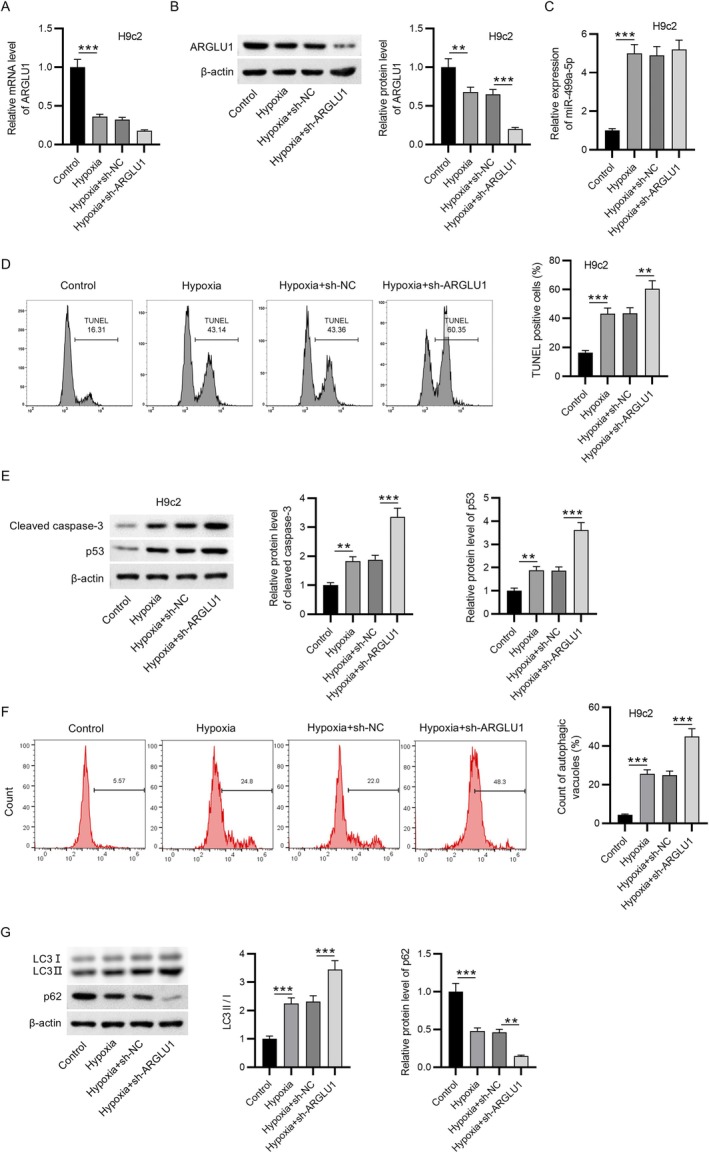
Knockdown of ARGLU1 promoted hypoxia‐induced cell apoptosis and autophagy. (A) RT‐qPCR analysis of ARGLU1 mRNA expression was performed in H9c2 cells under control, hypoxia, hypoxia + sh‐NC, and hypoxia + sh‐ARGLU1 groups. (B) ARGLU1 protein levels were quantified by western blotting in the indicated groups. (C) RT‐qPCR was conducted to evaluate miR‐499a‐5p expression in the same experimental conditions. (D) TUNEL assay was used to determine apoptotic levels in H9c2 cells following hypoxia and/or ARGLU1 knockdown. (E) Protein levels of cleaved caspase‐3 and p53 in H9c2 cells were quantified by western blotting. (F) Flow cytometry analysis was conducted to measure H9c2 cell autophagy. (G) Protein expression of autophagy‐related markers (LC3I, LC3II, and p62) was evaluated by western blotting. Data are expressed as mean ± SEM. **p < 0.01, ***p < 0.001.

To further explore the functional role of ARGLU1 under hypoxic conditions, its expression was silenced in H9c2 cells via transfection with sh‐ARGLU1. As shown in Figure 5A,B, both mRNA and protein levels of ARGLU1 were significantly reduced in the hypoxia + sh‐ARGLU1 group compared to the hypoxia + sh‐NC group, confirming effective knockdown. Importantly, ARGLU1 silencing did not influence the hypoxia‐induced upregulation of miR‐499a‐5p (Figure 5C). Functionally, the percentage of apoptotic H9c2 cells was markedly elevated under hypoxic conditions and was further increased upon ARGLU1 knockdown (Figure 5D). This was accompanied by enhanced expression of the apoptotic markers cleaved caspase‐3 and p53, which were already upregulated under hypoxia and further intensified by ARGLU1 depletion (Figure 5E). In addition, ARGLU1 silencing significantly exacerbated hypoxia‐induced autophagy, as evidenced by increased autophagic vacuole accumulation (Figure 5F). Correspondingly, there was a notable increase in the LC3‐II/LC3‐I ratio and a further reduction in p62 protein levels, indicating enhanced autophagic flux(Figure 5G). These findings suggest that ARGLU1 acts as a negative regulator of hypoxia‐induced apoptosis and autophagy in H9c2 cardiomyocytes, and that its knockdown amplified cellular injury under hypoxic stress.

### 3.6 ARGLU1 Knockdown Reversed the Suppressive Effect of miR‐499a‐5p Knockdown on Hypoxia‐Stimulated H9c2 Cell Injury

**FIGURE 6:**
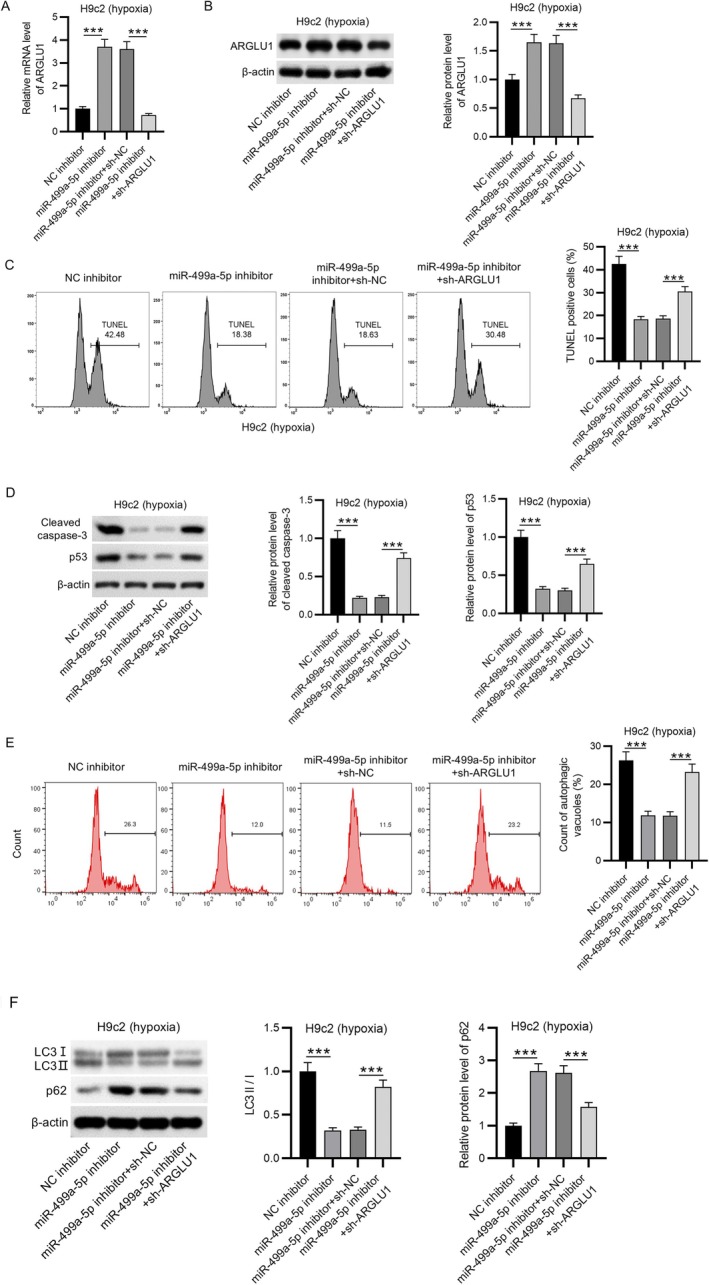
ARGLU1 knockdown reversed the suppressive effect of miR‐499a‐5p knockdown on hypoxia‐stimulated H9c2 cell injury. (A–B) RT‐qPCR and western blotting RT‐qPCR and western blot analyses were performed to assess ARGLU1 mRNA and protein expression in H9c2 cells treated with NC inhibitor, miR‐499a‐5p inhibitor, miR‐499a‐5p inhibitor + sh‐NC, and miR‐499a‐5p inhibitor + sh‐ARGLU1. (C) TUNEL assays were conducted to evaluate apoptosis in hypoxia‐exposed H9c2 cells following miR‐499a‐5p inhibition and/or ARGLU1 knockdown. (D) Protein levels of cleaved caspase‐3 and p53 were quantified by western blotting in hypoxia‐stimulated H9c2 cells. (E) Autophagy levels were assessed by flow cytometry in H9c2 cells subjected to hypoxia and differential regulation of miR‐499a‐5p and ARGLU1. (F) Western blot analysis was used to measure the expression of autophagy‐related proteins LC3I, LC3II, and p62. Data are presented as mean ± SEM.***p < 0.001.

To determine whether miR‐499a‐5p regulates hypoxia‐induced apoptosis and autophagy in H9c2 cells through targeting ARGLU1, a series of rescue experiments were conducted. As shown in Figure 6A,B, inhibition of miR‐499a‐5p significantly upregulated both mRNA and protein levels of ARGLU1, while this upregulation was reversed by ARGLU1 knockdown via sh‐ARGLU1 co‐transfection. Functionally, miR‐499a‐5p inhibition led to a marked reduction in H9c2 cell apoptosis, an effect that was rescued by ARGLU1 knockdown (Figure 6C). Similarly, the downregulation of cleaved caspase‐3 and p53 protein levels observed following miR‐499a‐5p inhibition was partially restored by sh‐ARGLU1, indicating that ARGLU1 mediates the anti‐apoptotic effects of miR‐499a‐5p suppression (Figure 6D). Moreover, the formation of autophagic vacuoles was suppressed by the inhibition of miR‐499a‐5p expression, and the alteration was rescued by ARGLU1 knockdown (Figure 6E). Furthermore, the decrease in LC3‐II/LC3‐I ratio and the increase in p62 levels, thus reflecting suppressed autophagy due to miR‐499a‐5p inhibition, were reversed upon ARGLU1 silencing (Figure 6F). Collectively, these findings demonstrate that miR‐499a‐5p can promote hypoxia‐induced apoptosis and autophagy in H9c2 cardiomyocytes primarily by downregulating ARGLU1, highlighting the miR‐499a‐5p/ARGLU1 axis as a potential therapeutic target in MI.

## 4 Discussion

The incidence of MI has been rising, particularly among younger individuals, largely due to elevated psychosocial stress levels in modern life [19]. In recent years, miRNAs have emerged as a major focus in MI research for their ability to regulate key cellular processes such as autophagy and apoptosis [20]. Among them, miR‐499a‐5p has been implicated in diverse biological functions, including cortical neuron apoptosis [21], cancer cell proliferation and migration [22], and the regulation of extracellular matrix homeostasis through modulation of anabolic and catabolic signaling [23]. Importantly, miR‐499a‐5p has demonstrated cardioprotective effects in both H9c2 cells exposed to hypoxia/reoxygenation and AC16 human cardiomyocytes treated with lipopolysaccharide [15, 16]. Furthermore, miR‐499a‐5p has been closely associated with cardiovascular pathologies and is recognized as a potential biomarker for acute MI [17, 24]. Cardiac remodeling, characterized by alterations in ventricular size and function, is a common consequence of heart diseases such as MI and hypertension [25]. In our study, an MI mouse model was successfully established, displaying significant cardiac fibrosis and functional impairment in comparison to sham‐operated controls. Additionally, miR‐499a‐5p expression was markedly elevated in the cardiac tissues of MI mice and similarly upregulated in hypoxia‐stimulated H9c2 cells, supporting the involvement of miR‐499a‐5p in MI pathogenesis and progression.

During MI, a substantial proportion of cardiomyocytes undergo cell death through various mechanisms, including necrosis, apoptosis, necroptosis, autophagy‐associated cell death, pyroptosis, and ferroptosis [26]. In our study, knockdown of miR‐499a‐5p significantly attenuated apoptosis in hypoxia‐induced H9c2 cells. Apoptosis in cardiomyocytes is regulated by several pro‐apoptotic factors, such as Bax and caspase‐3, while p53 plays a pivotal role as an apoptosis‐inducing transcription factor in the heart [27]. Consistent with this, our results demonstrated that miR‐499a‐5p inhibition led to a notable reduction in cleaved caspase‐3 and p53 protein levels under hypoxic conditions. Although autophagy typically serves as a protective mechanism in cardiomyocytes, excessive or dysregulated autophagy has been shown to exert detrimental effects on cardiac structure and function [28]. Previous studies have confirmed that autophagy is activated in various animal and cellular models of MI and that modulating autophagy can serve as a therapeutic strategy for mitigating MI [29, 30, 31]. In our current study, hypoxia stimulation resulted in increased accumulation of autophagic vacuoles, an elevated LC3‐II/LC3‐I ratio, and reduced p62 expression, all indicative of excessive autophagic activity in H9c2 cells. However, miR‐499a‐5p knockdown significantly reduced autophagic vacuole formation, decreased the LC3‐II/LC3‐I ratio, and upregulated p62 protein levels, suggesting a suppression of autophagic flux. Taken together, these findings indicate that inhibition of miR‐499a‐5p can effectively alleviate hypoxia‐induced apoptosis and autophagy in H9c2 cardiomyocytes.

It is well‐established that microRNAs (miRNAs) regulate gene expression by inhibiting mRNA translation or promoting mRNA degradation [32]. In our study, ARGLU1 was identified and experimentally confirmed as a direct downstream target of miR‐499a‐5p. Although ARGLU1 has been shown to regulate various cellular processes such as cell proliferation, cell cycle progression, apoptosis, and extracellular matrix degradation [33, 34], its role in cardiovascular diseases remains largely unexplored. Our findings revealed that ARGLU1 expression was significantly downregulated in the cardiac tissues of MI mice compared to sham‐operated controls. Similarly, a marked reduction in ARGLU1 levels was observed in H9c2 cells exposed to hypoxic conditions. Importantly, ARGLU1 silencing further enhanced hypoxia‐induced apoptosis and autophagy in H9c2 cells. Moreover, knockdown of ARGLU1 abolished the protective effects conferred by miR‐499a‐5p inhibition, reversing the suppression of apoptosis and autophagy in hypoxia‐treated H9c2 cells. These results suggest that ARGLU1 can mediate the regulatory effects of miR‐499a‐5p, and its downregulation contributes to the exacerbation of MI under hypoxic conditions.

In conclusion, our findings reveal that miR‐499a‐5p knockdown alleviates hypoxia‐induced H9c2 cell injury by suppressing apoptosis and autophagy through the upregulation of ARGLU1 expression. These findings uncover a previously underexplored miR‐499a‐5p/ARGLU1 regulatory axis, offering new insight into the molecular mechanisms underlying myocardial injury and identifying miR‐499a‐5p as a potential therapeutic target for MI. The results provide strong preclinical evidence supporting the therapeutic value of miR‐499a‐5p inhibition in protecting cardiomyocytes under ischemic stress. However, the in vivo effects of miR‐499a‐5p inhibition on myocardial fibrosis and cardiac dysfunction following MI were not explored in this study. To comprehensively assess its therapeutic potential, future investigations should incorporate additional experimental groups to determine whether miR‐499a‐5p suppression confers cardioprotective effects in MI preclinical models. Furthermore, the downstream signaling pathways modulated by ARGLU1 remain largely unknown and warrant further in‐depth investigation to clarify its role in cardiomyocyte homeostasis and myocardial injury. Overall, our study lays the groundwork for novel miRNA‐based therapeutic strategies targeting miR‐499a‐5p and ARGLU1 in the treatment of ischemic heart disease. 

## Ethics Statement

All experimental procedures involving animals were approved by the Institutional Animal Care and Use Committee of Hubei Provincial Center for Disease Control and Prevention (Safety Evaluation Research Center Animal Welfare No. 202320113; July 12th, 2023).

## Conflicts of Interest

The authors declare no conflicts of interest.

## Data Availability

The data that support the findings of this study are available from the corresponding author upon reasonable request.
